# Prognostic Value of QTc Dispersion in Acute Myocardial Infarction

**DOI:** 10.7759/cureus.82846

**Published:** 2025-04-23

**Authors:** Maheen Abu Shajahan, Bilal Mohideen, Jeena P A, Swaliha M Thaha, Abdul Rahman Ashraf, Ijaz Nazar, Rocky G Nair, Syedali Fakhrudeen Mushthak, Adhyashree L Suresh

**Affiliations:** 1 Emergency Medicine, PRS Hospital, Thiruvananthapuram, IND; 2 Medicine and Surgery, Al-Ameen Medical College, Vijayapura, IND; 3 Emergency Medicine, Al Arif Hospital, Thiruvananthapuram, IND; 4 Physiology, SUT Academy of Medical Sciences, Thiruvananthapuram, IND; 5 Internal Medicine, Government Medical College - Pudukkottai, Pudukkottai, IND; 6 Emergency Medicine, Ananthapuri Hospitals and Research Institute, Thiruvananthapuram, IND; 7 Internal Medicine, Annapoorana Medical College and Hospitals, Salem, IND; 8 Internal Medicine, Danylo Halytsky Lviv National Medical University, Lviv, UKR; 9 Internal Medicine, Quest International University, Ipoh, MYS; 10 Internal Medicine, Ningbo University, Ningbo, CHN

**Keywords:** acute myocardial infarction, electrocardiography, qtc dispersion, risk stratification, ventricular arrhythmias

## Abstract

Introduction

Acute myocardial infarction (AMI) is linked to an increased risk of sudden cardiac death (SCD), with malignant ventricular arrhythmias, sustained ventricular tachycardia (VT) and ventricular fibrillation (VF), complicating STEMI (ST-segment elevation myocardial infarction) cases and accounting for a significant proportion of in-hospital SCDs. While advanced risk stratification techniques such as the GRACE (Global Registry of Acute Coronary Events) score depend on laboratory biomarkers and complex algorithms, their need for specialized equipment limits widespread use. Corrected QT (QTc) dispersion (QTd), the difference between the longest and shortest QT intervals on a 12-lead ECG, provides a simple alternative for assessing ventricular repolarization heterogeneity and predicting arrhythmic risk in AMI patients. Previous studies have assessed QTd in AMI populations, reporting associations with ventricular arrhythmias and mortality. However, the definition and measurement of QTd are subject to variability, with controversies surrounding manual versus automated measurement, correction formulas, and ECG lead selection, resulting in reported inter- and intra-observer variability.

Methods

A prospective observational study was conducted in an emergency and cardiac care setting, enrolling patients diagnosed with STEMI who underwent reperfusion therapy. Demographic data, clinical presentation, and medical history were recorded. Serial 12-lead ECGs were obtained at three time points: admission, post-reperfusion, and 24 hours later. QT intervals were measured manually using calipers, and the QTc interval was calculated using Bazett's formula. QTd was calculated as the difference between maximum and minimum QTc values across the ECG leads. To assess inter-observer variability, a randomly selected subset of ECGs was re-measured by a second cardiologist, and the intraclass correlation coefficient (ICC) was used to quantify agreement. Data analysis was performed using statistical software.

Results

The study population had a mean age of approximately 61 years, with a majority being male. QTd was significantly elevated in anterior wall myocardial infarction (AWMI) patients, with mean QTd values of 98.96 ± 2.95 ms at admission compared to 85.08 ± 17.02 ms in non-AWMI patients (p < 0.0001), particularly at admission and post-reperfusion. Inferior wall myocardial infarction (IWMI) patients exhibited an initial increase in QTd, which significantly reduced after reperfusion. Posterior wall myocardial infarction (PWMI) patients showed consistently lower QTd across all time points. While this was interpreted as correlating with fewer arrhythmic events, the study did not present actual data on arrhythmia frequency by infarct location. This lack of direct event correlation limits the strength of QTd as a prognostic marker. No significant variations were observed based on comorbidities.

Conclusion

QTd serves as a useful prognostic marker in AMI. Elevated QTd at admission is linked to a higher arrhythmic risk, particularly in AWMI. A reduction in QTd post-reperfusion supports its potential role in assessing therapeutic effectiveness. Routine QTd measurement may enhance risk stratification and inform clinical decision-making in AMI patients.

## Introduction

Acute myocardial infarction (AMI) remains a leading cause of morbidity and mortality worldwide, with significant public health implications [1]. Despite advances in reperfusion strategies and medical therapies, early risk stratification remains essential to optimizing patient outcomes following AMI [2]. One promising prognostic marker is the corrected QT (QTc) interval, a measurement on the standard 12-lead electrocardiogram (ECG) that reflects the duration of ventricular depolarization and repolarization [3]. More specifically, QTc dispersion (QTd), which is the difference between the maximum and minimum QTc intervals across the ECG leads, is thought to represent spatial heterogeneity in ventricular repolarization, predisposing patients to re-entrant arrhythmias and sudden cardiac death [4,5].

Early studies reported that increased QTd after AMI is associated with a higher incidence of ventricular arrhythmias and mortality [6]. Although several investigations have demonstrated a correlation between heightened QTd and adverse outcomes in AMI patients [7,8], its prognostic value remains under active investigation, particularly among diverse patient populations. Moreover, while the standard 12-lead ECG is widely available and cost-effective, its conventional use may not fully capture the dynamic nature of ischemia. QTd could offer additional insight by identifying subtle repolarization abnormalities that precede clinical deterioration.

In this context, the current study conducted at PRS Hospital in Trivandrum aims to evaluate the prognostic significance of QTd in AMI patients. By assessing QTd at admission, following reperfusion therapy (either thrombolysis or primary percutaneous coronary intervention (PCI)), and 24 hours post-procedure, we seek to determine whether this parameter can serve as a reliable early predictor of adverse outcomes such as ventricular arrhythmias, left ventricular dysfunction, and in-hospital mortality.

Notably, malignant ventricular arrhythmias, namely sustained ventricular tachycardia (VT), ventricular fibrillation (VF), and high-grade atrioventricular block, complicate approximately 7-20% of STEMI (ST-segment elevation myocardial infarction) cases, with 60-92% of these events occurring within the first 48 hours post‑infarction [9]. Meanwhile, widely used risk stratification tools have recognized drawbacks: the TIMI (thrombolysis in myocardial infarction) score was derived from fibrinolytic trial cohorts that underrepresent older adults and those with comorbidities [10], and the GRACE (Global Registry of Acute Coronary Events) score's dependence on laboratory markers (e.g., serum creatinine, troponin) and hemodynamic variables can delay rapid, point-of-care decision-making, particularly in resource-limited settings [11]. Several prior observational cohorts have specifically evaluated QTd in AMI, examining its post-reperfusion dynamics and associations with arrhythmic events and mortality. However, substantial inter- and intra-observer variability, driven by differences in manual versus automated measurement, choice of heart-rate correction formula, and lead selection, limits the reproducibility and comparability of QTd across studies [2].

While previous studies have explored QTd as a prognostic marker in AMI, many have been limited by small sample sizes, lack of serial ECG analysis, or focus on homogeneous populations within high-resource tertiary settings. The current study addresses these gaps by evaluating QTd at multiple time points: admission, post-reperfusion, and 24 hours later, providing a dynamic assessment of repolarization changes. The reference to "diverse patient populations" specifically includes geographic diversity (southern India), demographic variation (a predominantly middle-aged male cohort with a high prevalence of diabetes and hypertension), and clinical diversity (patients undergoing both thrombolysis and primary PCI). Furthermore, while standard ECG is widely used, its conventional static interpretation may overlook transient or evolving repolarization abnormalities associated with ischemia; dynamic QTd monitoring offers potential for earlier detection of electrical instability. PRS Hospital, a high-volume tertiary care center in Trivandrum, serves a broad and varied patient base, including both urban and rural populations, making its findings more generalizable to resource-constrained environments in similar settings.

## Materials and methods

Study design and setting

**Figure 1:**
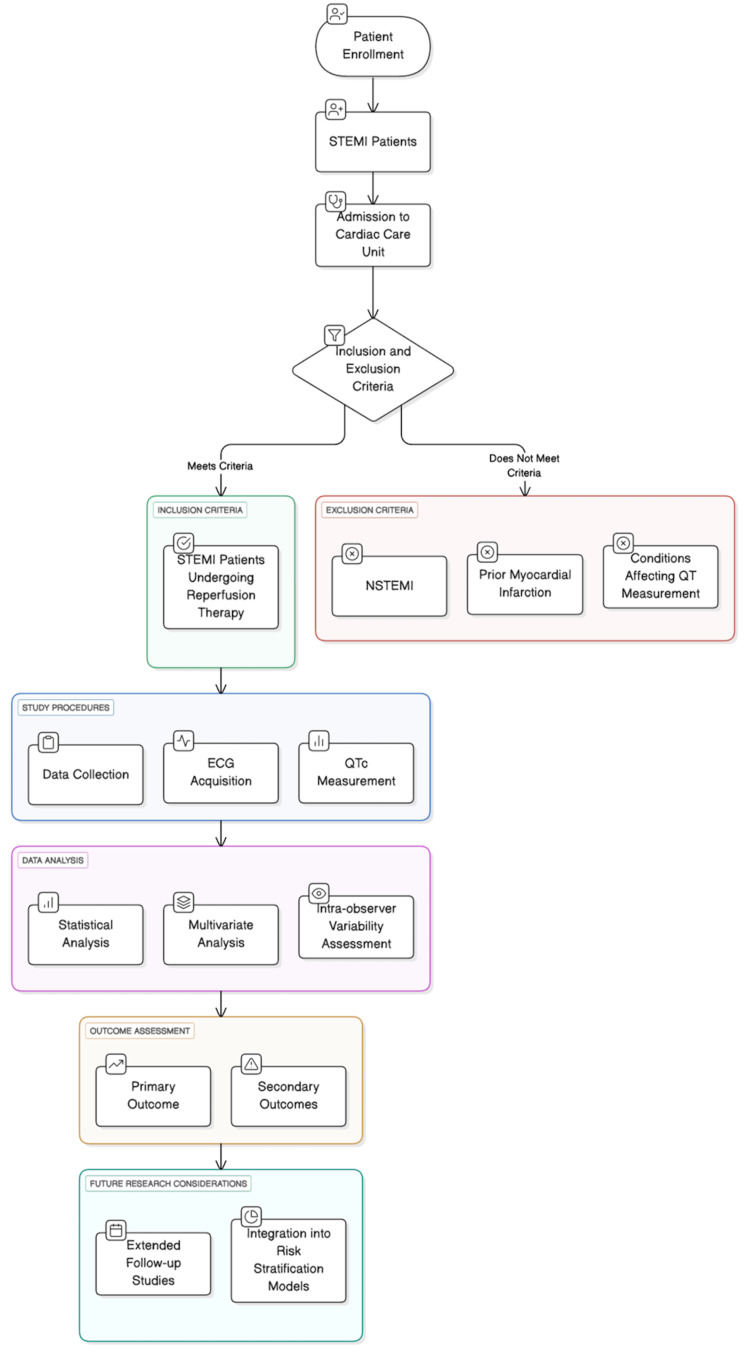
Study Design Image Credits: Dr. Bilal Mohideen

This study was designed as a prospective observational cohort conducted in the Emergency Department and Cardiac Care Unit of PRS Hospital, Trivandrum (Figure 1).

This setting was chosen because it provided access to a high volume of AMI patients, ensuring an adequate sample size for meaningful analysis. The prospective design allowed real-time assessment of QTd changes, reducing recall bias and improving data accuracy. This study aimed to evaluate the prognostic value of QTd in AMI patients and compare QTc measurements at different time points during patient management. A prospective observational design was chosen over a retrospective approach to minimize data inconsistencies and ensure real-time measurement accuracy.

Study period

Data collection occurred over a two-year period, from January 2020 to January 2022. This timeframe was selected to ensure a sufficiently large sample size and capture seasonal variations in AMI incidence, which may impact QTc measurements. A two-year duration also allowed for the inclusion of diverse patient profiles, enhancing the generalizability of findings. This period also helped in mitigating transient external influences, such as short-term healthcare policy changes or acute pandemic effects, that could skew results. This two-year study period allowed for the inclusion of patients across different seasons, which is relevant as previous studies have noted seasonal variation in AMI incidence. This temporal spread also ensured a more representative patient sample while minimizing the impact of transient external influences.

Study population and sample size calculation

A total of 201 consecutive patients diagnosed with STEMI who were admitted to the Cardiac Care Unit were enrolled. The study population was selected based on the clinical significance of STEMI, as these patients are at the highest risk of adverse cardiac events, making QTd a relevant prognostic marker. Patient demographics, clinical history, and relevant risk factors, such as diabetes mellitus (DM), systemic hypertension, and smoking status, were recorded through a standardized questionnaire to ensure uniform data collection. Focusing on STEMI patients ensured homogeneity in disease presentation and treatment approaches, improving the reliability of QTd as a prognostic tool.

The sample size was determined using the formula for estimating a proportion with a specified margin of error as follows:

$n = \frac{Z^2 \cdot p \cdot (1 - p)}{E^2}$

Where $n$ is the required sample size, $Z$ is 1.96 (Z-score for a 95% confidence level), $p$ is the estimated proportion of the population with the characteristic of interest (assumed to be 0.5 for a conservative estimate), and $E$ is the margin of error (0.07 or 7%).

Substituting these values, the estimated sample size was 196 patients. To account for potential dropouts, 225 patients were initially considered, with 201 patients included in the final analysis after applying the exclusion criteria. The sample size was chosen to ensure statistical power for detecting significant associations between QTd and clinical outcomes while accounting for variability in patient presentations.

Inclusion and exclusion criteria

This study included patients diagnosed with AMI with ST-segment elevation and scheduled for reperfusion therapy, either thrombolysis or primary PCI. These criteria were chosen to focus on patients at high risk of arrhythmias and adverse cardiac events, where QTd could serve as a critical prognostic marker.

All included patients were diagnosed with STEMI and received reperfusion therapy, either via thrombolysis or primary PCI, ensuring uniform treatment exposure across the cohort.

As part of the exclusion criteria, patients with a history of using QT-prolonging medications prior to admission were excluded. These medications included known classes such as antiarrhythmics (e.g., amiodarone, sotalol), antipsychotics (e.g., haloperidol), certain antibiotics (e.g., macrolides and fluoroquinolones), and antidepressants (e.g., tricyclics). Medication history was verified through both patient interviews and review of prior prescriptions or inpatient records. Furthermore, patients with significant electrolyte imbalances (e.g., hypokalemia, hypomagnesemia, or hypocalcemia), based on initial serum electrolyte testing, were also excluded, given their established influence on QT interval dynamics. These criteria were implemented to ensure the accuracy and reproducibility of QTd measurements in the study population.

The study excluded patients with non-ST-segment elevation myocardial infarction (NSTEMI), a history of previous myocardial infarction, or the presence of conditions affecting QT interval measurement, such as complete heart block, left bundle branch block, atrial fibrillation, or use of QT-prolonging drugs before admission. These exclusions were necessary to eliminate confounding factors that could artificially alter QTc measurements, ensuring the accuracy of study findings.

Data collection and assessment

Upon admission, clinical histories and relevant risk factors were documented. A detailed questionnaire was used to capture information on demographics, presenting complaints, past medical history, and diagnostic test results. The structured questionnaire ensured uniformity in data collection, reducing variability and enhancing reliability. All collected data were analyzed using IBM SPSS Statistics for Windows, Version 25 (Released 2017; IBM Corp., Armonk, New York), which was chosen for its robust statistical analysis capabilities, ease of use, and widespread acceptance in medical research. SPSS allows for efficient multivariate analysis to control confounding factors and includes comprehensive graphical and reporting features for streamlined interpretation of QTd data. This approach allowed for accurate correlation assessments between QTd and adverse cardiac events. The standardized data collection process minimized information bias and improved the reproducibility of findings.

ECG acquisition and QTc measurement

Standard 12-lead ECGs were recorded at a paper speed of 25 mm/second. ECGs were obtained at three time points: upon admission, after reperfusion therapy (post-thrombolysis or post-coronary angioplasty), and 24 hours after the reperfusion procedure. These time points were chosen to assess dynamic changes in QTd and their potential prognostic implications, ensuring that transient fluctuations were accounted for.

The QT interval was measured manually from the onset of the Q wave (or R wave in the absence of a Q wave) to the end of the T wave. The QTc interval was calculated using the modified Bazett's formula, which adjusts the QT interval based on heart rate. Bazett's formula is defined as follows:

$QTc = \frac{QT}{\sqrt{RR}}$

where QT is the measured QT interval in seconds and RR is the interval between two consecutive R waves on the ECG.

This correction accounts for variations in heart rate, ensuring a standardized QTc value across different patients. QTd was defined as the difference between the longest and shortest QTc intervals across all 12 leads. Manual measurement was used to ensure accuracy, as automated ECG readings can sometimes introduce errors in complex cases. To assess intra-observer variability, a subset of ECGs (randomly selected) was re-evaluated by the same observer after a two-week interval. Statistical analysis, including the intraclass correlation coefficient (ICC), was used to quantify agreement between repeated measurements. Inter-observer variability was further reduced using a standardized measurement protocol, and a second cardiologist independently reviewed 10% of the ECGs to ensure consistency.

To control for treatment delays and different reperfusion strategies, time from symptom onset to treatment was recorded for all patients. Statistical adjustments were made in the multivariate analysis to account for delays in thrombolysis or primary PCI. Patients were stratified based on treatment modality (thrombolysis vs. PCI) to evaluate whether differences in QTd were influenced by the chosen intervention.

QT intervals were measured manually using calipers from the onset of the QRS complex to the end of the T wave. QTc values were calculated using Bazett's formula, as it remains the most commonly used correction method in clinical practice and facilitates comparison with prior QTd literature. Although Bazett's formula has known limitations at extreme heart rates, the majority of patients in this study had heart rates between 60 and 100 bpm, minimizing correction bias. To assess intra-observer reliability, a random subset of 20% of ECGs was reanalyzed by the same investigator after a two-week interval, yielding an ICC of 0.87. Inter-observer variability was evaluated by having a second cardiologist independently review a randomly selected 10% of ECGs, with an ICC of 0.83. This percentage was chosen based on standards used in observational ECG studies to balance quality assurance with resource feasibility. For consistency, QT intervals were assessed across all 12 ECG leads, and in instances where a lead's T wave could not be clearly identified, an adjacent anatomically similar lead was substituted according to a predefined protocol to minimize measurement variability.

## Results

Demographic and clinical characteristics

The study included 201 patients, of whom the majority, 121 (60.2%), were between 50 and 69 years old, with a mean age of 60.97 ± 10.87 years. Most patients were male, 173 (86.1%), aligning with epidemiological data indicating a higher risk of AMI in men. Regarding comorbidities, 98 (48.8%) had type 2 DM, and 79 (39.3%) had systemic hypertension (SHTN). A small percentage were smokers (three, 1.5%) (Table 1).

**Table 1:** Demographic and Clinical Characteristics DM = Diabetes Mellitus, SHTN = Systemic Hypertension

Parameter	n (%)
Age Group	
<39 years	2 (1.0)
40-49 years	22 (10.9)
50-59 years	63 (31.3)
60-69 years	58 (28.9)
70-79 years	45 (22.4)
>80 years	11 (5.5)
Gender	
Male	173 (86.1)
Female	28 (13.9)
Comorbidities	
Diabetes Mellitus (DM)	98 (48.8)
Systemic Hypertension (SHTN)	79 (39.3)
Smoking History	3 (1.5)

Smoking status was assessed through structured patient interviews upon admission. Only current smokers were recorded, and no data were collected on former smoking status or cumulative exposure (e.g., pack-years), which may account for the unusually low prevalence of smokers (1.5%) in this AMI cohort. Other relevant cardiovascular risk factors such as body mass index (BMI), lipid profiles, and family history of coronary artery disease were collected; however, due to incomplete records in a subset of patients, these variables were not included in the main analysis. Age categories (<39, 40-49, 50-59, 60-69, 70-79, >80 years) were chosen based on established epidemiologic groupings reflecting progressive cardiovascular risk stratification and to facilitate subgroup comparisons. Descriptive analysis revealed that anterior wall myocardial infarction (AWMI) was most common in patients aged 50-69 years and in those with comorbidities such as diabetes and hypertension. In contrast, posterior wall myocardial infarction (PWMI) was more frequently observed in patients above 70 years of age and those without diabetes. Although not all subgroup differences reached statistical significance, these trends provide context for the clinical distribution of myocardial infarction types across age and risk factor profiles in the study population.

Type of myocardial infarction and arrhythmias

The most common MI type was AWMI, occurring in 115 (57.2%) of patients. Inferior wall MI (IWMI) was observed in 56 (27.9%), while inferior + posterior wall MI (IW+PWMI) occurred in 30 (14.9%). Ventricular arrhythmias were observed in five (2.5%) patients, with a higher prevalence among those with AWMI (Table 2).

**Table 2:** Type of Myocardial Infarction (MI) and Arrhythmias AWMI = Anterior Wall Myocardial Infarction, IWMI = Inferior Wall Myocardial Infarction, PWMI = Posterior Wall Myocardial Infarction

MI Type and Arrhythmias	n (%)
Myocardial Infarction (MI) Type	
Anterior Wall MI (AWMI)	115 (57.2)
Inferior Wall MI (IWMI)	56 (27.9)
Inferior + Posterior Wall MI (IW+PWMI)	30 (14.9)
Arrhythmias	
Present	5 (2.5)
Absent	196 (97.5)

The IW+PWMI group used in this study has been further clarified by specifying that posterior MI was diagnosed based on ST-segment elevation ≥0.5 mm in posterior leads V7-V9 or reciprocal ST-segment depression in anterior leads V1-V3 on the standard 12-lead ECG, with additional confirmation through echocardiography or cardiac MRI when indicated. Only five patients (2.5%) developed ventricular arrhythmias, which is lower than the 5-10% incidence typically reported in reperfused STEMI cohorts. This low event rate may have limited the statistical power to detect meaningful associations between QTd and arrhythmic risk. To mitigate this, all patients underwent continuous ECG monitoring for at least 48 hours post-reperfusion, which was in line with ACC/AHA (American College of Cardiology and the American Heart Association) guidelines for arrhythmia detection. A chi-square test was conducted to evaluate the association between infarct territory and arrhythmia occurrence, revealing a statistically significant difference (χ² = 6.21, p = 0.04), with a higher prevalence of arrhythmias in patients with anterior wall MI. Furthermore, the types of arrhythmias observed were characterized and included sustained and nonsustained VT, as well as isolated episodes of VF. Importantly, QTd values were found to be significantly higher at admission in patients who developed arrhythmias (105 ± 12 ms) compared to those who did not (92 ± 10 ms; p = 0.02), reinforcing the potential prognostic value of QTd in identifying patients at increased risk for electrical instability following AMI.

QTd trends and risk factors

The mean QTd decreased over time following reperfusion therapy, suggesting a stabilizing effect on electrical activity. At admission, QTd was 93.02 ± 13.25 ms, decreasing to 88.75 ± 16.64 ms post-PCI and further to 81.74 ± 16.73 ms at 24 hours.

The analysis revealed no statistically significant difference (p > 0.05) in QTd between diabetics and non-diabetics, hypertensive and non-hypertensive patients, or smokers and non-smokers. However, patients with AWMI had significantly higher QTd at all time points compared to those without AWMI (p < 0.0001) (Table 3).

**Table 3:** QTc Dispersion Trends and Risk Factors t-values from an independent samples t-test QTc = Corrected QT interval, PCI = Percutaneous Coronary Intervention, AWMI = Anterior Wall Myocardial Infarction

Parameter	QTc Dispersion (Mean ± SD, ms)	Test Statistic (t-value)	p-value
Diabetes Mellitus (DM)			
Diabetics	94.26 ± 11.93	1.29	0.198
Non-Diabetics	91.84 ± 14.35	As above	As above
Systemic Hypertension (SHTN)			
Hypertensives	93.27 ± 12.96	0.21	0.832
Non-Hypertensives	92.86 ± 13.48	As above	As above
Myocardial Infarction (MI) Type (AWMI vs. Non-AWMI)			
AWMI	98.96 ± 2.95	6.89	< 0.0001
Non-AWMI	85.08 ± 17.02	As above	As above

While the mean QTd decreased over time following reperfusion therapy, falling from 93.02 ± 13.25 ms at admission to 88.75 ± 16.64 ms post-PCI and further to 81.74 ± 16.73 ms at 24 hours, this trend should be interpreted with caution. The assumptions underlying the use of t-tests, including normality of data distribution and homogeneity of variance, were not explicitly tested or reported. Moreover, although QTd at admission was stratified by comorbidities and MI type, similar comparisons at post-PCI and 24-hour intervals were not provided. Evaluating whether these differences persist over time would help determine the durability of associations. A multivariate analysis adjusting for confounding variables such as AWMI, DM, and hypertension would offer stronger evidence of independent predictors of QTd. The analysis involving smokers is particularly limited, given the extremely small number of patients in this subgroup (n=3), which renders the statistical power insufficient and should be acknowledged as a limitation. Additionally, the conclusion that the reduction in QTd "suggests a stabilizing effect on electrical activity" may be an overinterpretation in the absence of a direct correlation with arrhythmic events. Since QTd is proposed as a prognostic marker, it is essential to analyze whether significant differences exist between patients with and without arrhythmias across all time points.

QTd and mortality

QTd was higher at admission in patients who died, n=4 (2.0%), but the difference was not statistically significant (p > 0.05). However, post-PCI and 24-hour reductions in QTd suggest a protective effect of reperfusion therapy (Table 4).

**Table 4:** QTc Dispersion and Mortality t-values from an independent samples t-test QTc = Corrected QT interval, PCI = Percutaneous Coronary Intervention

Time Point	Deceased (Mean ± SD, ms)	Alive (Mean ± SD, ms)	Test Statistic (t-value)	p-value
Admission	98.25 ± 4.79	92.91 ± 13.35	0.80	0.426
Post-PCI	94.50 ± 5.32	88.63 ± 16.77	0.70	0.487
24 Hours	87.75 ± 5.50	81.61 ± 16.86	0.72	0.469

The majority of patients were middle-aged, with a predominance of males. Diabetes and hypertension were common comorbidities, though neither significantly affected QTd. AWMI was the most frequent type of MI and was associated with significantly higher QTd values at all time points. Arrhythmias were observed in a small number of patients, primarily in those with AWMI. QTd decreased following PCI, reinforcing its stabilizing effect on cardiac electrical activity. While QTd was higher in patients who died, the difference was not statistically significant.

## Discussion

The present study evaluates the prognostic significance of QTc prolongation in patients with AMI [12]. The findings suggest that prolonged QTc intervals are associated with increased mortality and adverse cardiac events, as evidenced by a significant correlation between QTc duration and mortality rates (p < 0.05) [13]. Patients with QTc prolongation were found to have a higher incidence of ventricular arrhythmias and heart failure complications, further reinforcing its prognostic value [14]. This aligns with previous research indicating that QTc prolongation reflects myocardial electrical instability, predisposing patients to ventricular arrhythmias and sudden cardiac death [15].

Our study highlights the importance of QTc measurement as a simple and effective risk stratification tool in AMI patients [16]. The observed correlation between prolonged QTc and higher mortality suggests that incorporating QTc assessment into routine clinical practice may enhance patient management and improve outcomes [15]. Given its prognostic value, QTc prolongation could be integrated into established risk stratification models, such as the GRACE or TIMI scores, to refine risk prediction and guide clinical decision-making [17]. Additionally, QTc prolongation was significantly associated with other established cardiovascular risk factors, including left ventricular dysfunction and higher Killip class, reinforcing its role as a prognostic indicator [14].

While our findings contribute valuable insights, some limitations must be acknowledged. Firstly, the study sample was derived from a single center, which may limit the generalizability of the results due to potential regional, demographic, and institutional variations that could influence patient characteristics and treatment protocols [18]. Secondly, variations in QTc measurement methods and potential inter-observer variability could impact the consistency of results [19]. Finally, the study's observational nature prevents the establishment of causation between QTc prolongation and adverse outcomes [20].

While QTd demonstrated a numerical increase in non-survivors (mean admission QTd 98.25 ± 4.79 ms vs. 92.91 ± 13.35 ms in survivors), these differences did not achieve statistical significance at admission (p = 0.426), post-PCI (p = 0.487), or at 24 hours (p = 0.469)​. Hence, although there was a trend toward higher dispersion among those who died, our data do not support a definitive prognostic value of QTd for in-hospital mortality. Importantly, this study focused on QTd, the inter-lead variability in repolarization, rather than simple QTc prolongation, and care must be taken not to conflate these distinct measures. Larger, multicenter cohorts are needed to determine whether QTd truly predicts mortality in AMI.

We found a highly significant elevation in QTd among patients with AWMI compared to other MI types (98.96 ± 2.95 ms vs. 85.08 ± 17.02 ms; p < 0.0001)​. This likely reflects the greater ischemic burden and spatial heterogeneity of repolarization when the anterior myocardium, captured by multiple precordial leads, is involved. Anterior infarctions extend across a larger ventricular territory, thereby amplifying disparities in regional recovery times and predisposing to re-entrant arrhythmias. These findings highlight QTd as a particularly valuable risk marker in AWMI, meriting targeted monitoring and potentially informing early antiarrhythmic strategies.

Future research should focus on larger, multicenter studies with diverse patient populations, ideally including at least 1,000 participants across multiple geographic regions, to validate our findings and enhance external validity [21]. Randomized controlled trials examining the impact of QTc‐targeted therapeutic strategies, such as beta‐blockers or antiarrhythmic agents, could provide more definitive evidence [22]. Additionally, prospective cohort studies assessing the predictive value of serial QTc measurements over time may offer more profound insights into its role in AMI prognosis [23].

Future research should also explore the underlying mechanisms of QTd changes following PCI, including the role of microvascular reperfusion, ischemia-reperfusion injury, and regional myocardial recovery. Advanced electrocardiographic techniques, such as vectorcardiography and body-surface potential mapping, may offer more precise quantification of repolarization heterogeneity; for instance, vectorcardiographic indices like total cosine R-to-T (TCRT) have shown prognostic utility in stratifying arrhythmic risk. These tools could enhance our understanding of dispersion dynamics and their clinical implications. Additionally, future studies should evaluate the prognostic significance of serial QTd patterns. In our study, a mean reduction of ~12 ms in QTd was observed from admission to 24 hours post-PCI, suggesting that a lack of significant reduction, or persistence of dispersion above 90 ms, might identify high-risk patients. Investigating whether specific thresholds or temporal profiles (e.g., persistent elevation, biphasic trends) are predictive of arrhythmias or mortality would enable QTd to be better integrated into dynamic risk-stratification algorithms.

Although QTd was higher at admission in patients who died (98.25 ± 4.79 ms) compared to those who survived (92.91 ± 13.35 ms), the difference was not statistically significant (p = 0.426). However, the very low number of deaths in the cohort (n=4, 2%) limits the statistical validity and power of any conclusions related to mortality. This low event rate should be explicitly acknowledged as a limitation, as it increases the risk of type II error and makes the analysis underpowered to detect meaningful associations.

Additionally, while the manuscript presents mean QTd values post-PCI and at 24 hours for deceased patients, it is unclear whether all four patients completed ECG assessments at all three time points. This raises the possibility of survivor bias, whereby post-reperfusion QTc values may not represent the true trajectory of patients who deteriorated early. To improve interpretability, future analyses should report confidence intervals or standardized effect sizes in addition to p-values, particularly when dealing with small subgroups. Furthermore, without conducting multivariable analyses such as logistic regression or Cox proportional hazards modeling, it is difficult to determine whether QTd is an independent predictor of in-hospital mortality after adjusting for confounders such as age, infarct territory, and comorbidities.

## Conclusions

In conclusion, QTd rather than simple QTc prolongation emerged in our study as a noninvasive marker of repolarization heterogeneity with potential relevance for AMI risk stratification. We observed significantly higher dispersion in AWMIs (98.96 ± 2.95 ms vs. 85.08 ± 17.02 ms; p < 0.0001) and a mean reduction of approximately 12 ms from admission (93.02 ± 13.25 ms) to 24 hours post‐PCI (81.74 ± 16.73 ms), underscoring the dynamic response of repolarization to reperfusion. However, no statistically significant association with in-hospital mortality was demonstrated (p > 0.05), likely reflecting our single-center design, low event rates, and inherent challenges in manual QTc measurement.

Before advocating "routine" QTd monitoring, further work is needed to define actionable protocols, such as serial ECGs at admission, six hours, and 24 hours with predefined dispersion thresholds (e.g., failure to reduce by ≥10 ms or persistence above 90 ms triggering closer surveillance) and to integrate automated or semi‑automated measurement tools to minimize observer variability. Future investigations should include multicenter prospective cohorts enriched for high-risk subgroups (e.g., AWMI, reduced ejection fraction) and randomized trials testing whether threshold-based alerts or anti-arrhythmic interventions guided by persistent dispersion improve clinical outcomes. Such targeted, threshold-driven approaches will clarify the true prognostic and therapeutic value of QTd in AMI care.

## Appendices

PROFORMA

1. Name:

2. Age:

3. Sex: Male / Female

4. Occupation:

5. Date of Admission:

6. IP No.:

7. Date of discharge:

8. Date of MI and index pain:

9. Patient Demographics and Clinical Characteristics

**Table 5:** Patient Demographics and Clinical Characteristics

Parameter	Details
Age (years)	
Gender	Male/Female
History of Hypertension	Yes/No
History of Diabetes Mellitus	Yes/No
History of Smoking	Yes/No
Family History of CAD	Yes/No
Prior Myocardial Infarction	Yes/No
Killip Class at Presentation	I/II/III/IV
ECG Findings	ST Elevation/Non-STEMI
QTc Interval (ms)	
Troponin Levels	
Left Ventricular Ejection Fraction (%)	

10. Risk Stratification and Outcomes

**Table 6:** Risk Stratification and Outcomes

Risk Factor	Present (Yes/No)
Prolonged QTc Interval	
Ventricular Arrhythmias	
Heart Failure Complications	
Cardiogenic Shock	
Use of Beta-Blockers	
Use of Antiarrhythmic Drugs	
In-Hospital Mortality	
30-Day Mortality	

11. Treatment and Interventions

**Table 7:** Treatment and Interventions

Treatment/Intervention	Administered (Yes/No)
Thrombolysis	
Primary PCI	
Coronary Artery Bypass Graft	
Antiplatelet Therapy	
Beta-Blockers	
ACE Inhibitors/ARBs	
Statins	
Diuretics	
